# Transgenerational Effects of a Neonicotinoid and a Novel Sulfoximine Insecticide on the Harlequin Ladybird

**DOI:** 10.3390/insects12080681

**Published:** 2021-07-28

**Authors:** Changchun Dai, Michele Ricupero, Zequn Wang, Nicolas Desneux, Antonio Biondi, Yanhui Lu

**Affiliations:** 1 State Key Laboratory for Biology of Plant Diseases and Insect Pests, Institute of Plant Protection, Chinese Academy of Agricultural Sciences, Beijing 100193, China; changchundai@163.com; 2 Department of Plant Protection, College of Agriculture, Northeast Agricultural University, Harbin 150030, China; 13503628686@163.com; 3 Langfang Experimental Station of the Chinese Academy of Agricultural Sciences, Langfang 065005, China; 4 Department of Agriculture Food and Environment, University of Catania, 95123 Catania, Italy; mricupero@unict.it (M.R.); antonio.biondi@unict.it (A.B.); 5 Université Côte d’Azur, INRAE, CNRS, UMR ISA, 06000 Nice, France; nicolas.desneux@sophia.inra.fr

**Keywords:** coccinellid, cotton aphid, IPM, neonicotinoid, sublethal effects, sulfoximine

## Abstract

**Simple Summary:**

The coccinellid *Harmonia axyridis* is an important natural enemy of various agricultural pests, including aphids. Agrochemicals can negatively affect the performance of arthropod natural enemies and, thus, the ecological services they provide. In this context, we assessed the lethal and sublethal effects of two neuroactive compounds with different chemical structures: the long-established neonicotinoid insecticide, imidacloprid, and the novel, sulfoximine insecticide, sulfoxaflor, both of which act on nicotinic acetylcholine receptors against adult and developmental stages of *H. axyridis*. Estimated LC_20_ and LC_50_ doses of imidacloprid for a target pest species, *Aphis gossypii*, resulted in significantly greater mortality in contact bioassays against adult *H. axyridis* compared with equivalent LC_20_ and LC_50_ doses of sulfoxaflor. Both concentrations of imidacloprid and sulfoxaflor significantly reduced the proportion of ovipositing females of parental generation. LC_20_ and LC_50_ dose of imidacloprid and LC_50_ dose of sulfoxaflor significantly reduced both the fecundity and fertility of parental generation. In progeny of parents exposed to both insecticides at LC_50_ concentrations the juvenile survival rate was significantly reduced, and both concentrations of imidacloprid and sulfoxaflor, except LC_20_ dose of sulfoxaflor, significantly prolonged the larval development time. These experimental results disclose the negative influence of sulfoxaflor and imidacloprid at low concentrations on the harlequin ladybird and its subsequent generation. Hence, actions should be taken to optimize imidacloprid and sulfoxaflor applications for the control of aphid pests, aiming at preserving the biocontrol services provided by this important predator.

**Abstract:**

The harlequin ladybird, *Harmonia axyridis* Pallas (Coleoptera: Coccinellidae), is a generalist predator and an effective biocontrol agent of various insect pests that has been exploited for the control of aphid pests in the greenhouse and field. However, insecticides are widely used to control aphid pests worldwide and the potential non-target effects of sulfoxaflor and imidacloprid for controlling aphid pests towards this biocontrol agent are little known. Although both sulfoxaflor and imidacloprid act on nicotinic acetylcholine receptors of insects, sulfoxaflor has a novel chemical structure compared with neonicotinoids. We assessed the lethal, sublethal and transgenerational effects of sulfoxaflor and imidacloprid on *H. axyridis* simultaneously exposed via ingestion of contaminated prey and via residual contact on the host plant at LC_20_ and LC_50_ doses estimated for the cotton aphid. Imidacloprid significantly reduced the survival of *H. axyridis* adults compared to sulfoxaflor at the same lethal concentration against cotton aphid. Both concentrations of imidacloprid and sulfoxaflor reduced the proportion of ovipositing females, and both concentrations of imidacloprid and sulfoxaflor, except LC_20_ dose of sulfoxaflor, reduced the fecundity and fertility of the parental generation. In the progeny of imidacloprid- and sulfoxaflor-exposed parents, both tested LC_50_ concentrations significantly decreased the juvenile survival rate, and both concentrations of imidacloprid and sulfoxaflor, except LC_20_ dose of sulfoxaflor, prolonged the development time. Our findings provide evidence of the negative influence of imidacloprid and sulfoxaflor at low lethal concentrations on the harlequin ladybird and on the progeny of exposed individuals, i.e., transgenerational effects. Hence, these findings stress the importance of optimizing the applications of imidacloprid and sulfoxaflor for the control of aphid pests, aiming at preserving the biocontrol services provided by *H. axyridis* throughout the integrated pest management approach.

## 1. Introduction

The harlequin ladybird, *Harmonia axyridis* Pallas (Coleoptera: Coccinellidae), is native to Asia, and since the last century it has been introduced into Europe and North America as a biological control agent against aphids and coccids [1,2,3]. *Harmonia axyridis* represents a key biological control agent for a variety of plant pests, with a broad dietary range and great capacity to suppress plant pests in both natural and agroecosystems [4,5,6,7]. For example, in cotton, abundant *H. axyridis* predators can suppress aphid populations below the economic threshold at seedling stages [8,9,10].

The cotton aphid, *Aphis gossypii* Glover (Hemiptera: Aphididae), a key pest of cotton, causes serious damage to cotton yield by sucking sap and transmitting viruses [11,12,13]. Although integrated pest management (IPM) programs have been implemented against *A. gossypii*, its management still primarily depends on the application of insecticides [14,15,16,17]. As a consequence, the overuse of pesticides can cause increasing resistance of primary pests, outbreaks of secondary pests and disruption of the ecosystem functioning and services for beneficial arthropods [18,19,20,21]. In China, *A. gossypii* has been controlled with neonicotinoids and more recently sulfoxamines in the past few years [18,22].

Over the last decades, imidacloprid has been used worldwide to control sap-sucking pests [23,24,25,26], and its use has increased significantly. However, long-term and extensive application of imidacloprid has led to increasing resistance of cotton aphids to imidacloprid [25,27]. Resistance to imidacloprid has been attributed in some cases to increased rates of insecticide detoxification or to mutations in nicotinic acetylcholine receptor (nAChR) [28,29]. Sulfoxaflor, a sulfoximine insecticide, has a proved efficacy for controlling a variety of sap-sucking pests and a recognized low toxicity towards mammals [30,31,32,33,34]. Although the actions of sulfoxaflor and imidacloprid were characterized at nAChRs of insect, sulfoxaflor has a limited cross-resistance towards neonicotinoid resistant pests due to its novel chemical structure [31,35,36]. For this reason, and the widespread occurrence of cotton aphid resistance to imidacloprid, sulfoxaflor is being increasingly used for the control of resistant cotton aphids [37]. By contrast, imidacloprid is suspected to lead to the disruption of ecological services and environmental pollution [38]. A plethora of studies in unison recognized the negative impact of neonicotinoid insecticides on natural enemies and pollinators even at low doses [39,40,41,42,43,44,45]. As a consequence, three neonicotinoid insecticides (i.e., imidacloprid, clothianidin and thiamethoxam) were recently banned for outdoor application in Europe in 2019 [46].

The evaluation of non-target effects of insecticides on beneficial arthropods generally includes both lethal and sublethal effects [39]. The former studies the acute toxicity and gives immediate feedback on the direct mortality caused by the pesticide [47]. The latter considers the physiological and behavioral impairments caused by the chemical to the non-target organisms, which may induce the reduction of their ecological services [48]. Coccinellid predators can be exposed to pesticides by direct contact with spray droplets and/or foliar residues of neonicotinoid insecticide when they are foraging on the crop, as well as by consuming the contaminated diet when feeding on food (e.g., pollen, prey) in the field [18,49,50,51,52,53,54]. Thus, the effects of pesticides at low dose/concentration on natural enemies frequently occurs after pesticide applications in the field [55,56,57,58].

To the best of our knowledge, several studies on acute toxicity of imidacloprid and sulfoxaflor to non-target natural enemies such as ladybeetle predators have been conducted [49,59]. However, the potential long-term influence of imidacloprid and sulfoxaflor on *H. axyridis* has been scarcely investigated [59]. Hence, we have explored the transgenerational impact of low lethal concentrations of sulfoxaflor and imidacloprid on *H. axyridis*. The results are expected to provide a valuable reference for optimizing the use of sulfoxaflor and imidacloprid as an effective component of IPM programs in agricultural settings.

## 2. Materials and Methods

### 2.1. Biological Materials

*Aphis gossypii* and *H. axyridis* laboratory colonies were originally obtained from infested cotton plants collected in cotton fields at the Langfang Experimental Station of the Chinese Academy of Agricultural Sciences (CAAS), Hebei Province, China, during summer in 2017.

The cotton aphid was continuously reared in screened cages (50 cm × 50 cm × 50 cm) on cotton plants (cv. “Zhongmian49”) in the laboratory at 24 ± 2 °C, 50 ± 10% RH, and L16:D8 photoperiod. The colony of *A. gossypii* was maintained in the laboratory for more than twenty generations before being used in the experiments. Cotton plants were grown in plastic pots (13 cm high, 15 cm diameter) with standard potting soil and then enclosed in cages without pesticides treatment during the plant growth period. Cotton plants at the five-leaf stage were used for cotton aphid rearing as well as for all the experiments.

Individuals of *H. axyridis* were reared on *Megoura japonica* Matsumura (Hemiptera: Aphididae), which were fed on pesticide-free seedlings of broad beans, *Vicia faba* L., at 20 ± 2 °C, 50 ± 10% RH, and L12:D12 photoperiod. The aphid-infested seedings of broad beans were offered to *H. axyridis* every two days within a fine mesh net plastic cage. Broad beans were cultivated in plastic pots. The laboratory conditions were the same as described above for the rearing of *A. gossypii*.

### 2.2. Chemicals

Commercial formulations of sulfoxaflor and imidacloprid commonly used for the control of cotton aphid were first tested to evaluate the baseline toxicity against *A. gossypii* laboratory colony. The concentrations causing the 20% (LC_20_) and 50% (LC_50_) mortality of *A. gossypii* were estimated (see Section 2.3), and these concentrations were then used to assess the lethal and sublethal effects on *H. axyridis*. Full information on the pesticides used in this experiment is summarized in Table 1. All tested pesticides were supplied by manufacturers in China.

### 2.3. Insecticide Baseline Toxicity on Prey

The dipping method [60] was used for assessing the concentration-mortality response of sulfoxaflor and imidacloprid on *A. gossypii*. Six serial concentrations of each insecticide formulation (3.50 ppm, 7.00 ppm, 14.00 ppm, 35.00 ppm, 70.00 ppm and 140.00 ppm for imidacloprid; 2.20 ppm, 5.50 ppm, 11.00 ppm, 22.00 ppm, 55.00 ppm and 110.00 ppm for sulfoxaflor) were diluted with distilled water for the bioassay. To obtain coetaneous *A. gossypii* young adults, 3-day-old nymphs of *A. gossypii* were collected from the rearing cage and moved to clean and uninfested cotton plants with a fine paint brush. After 7 days, excised cotton leaves bearing 20 coetaneous (24–48 h old) young adults *A. gossypii* were immersed in one of six pesticide solutions for 5 s and then were allowed to dry in laboratory conditions for 1 h. The control group was treated with distilled water. Each insecticide-concentration and the control were replicated five times, and cotton leaves per treatment with 20 young adults of *A. gossypii* were placed in individual Petri dishes (contain agar plate), which were covered with perforated PVC film. These Petri dishes were kept for 48 h in climatic chambers. *A. gossypii* mortality was scored by counting the number of surviving individuals under a stereomicroscope after the beginning of the exposure. The individuals were recorded as dead when they failed to crawl when pushed with a fine brush.

All the experiments were carried out under controlled environmental conditions at 25 ± 1 °C, 70 ± 5% RH, L16:D8 photoperiod and light intensity of 24,000 Lx in climatic chambers (RXZ-500D, Jiangnan Instrument Factory, Ningbo, China).

### 2.4. Lethal Toxicity on Harmonia Axyridis

We estimated the acute toxicity of LC_20_ and LC_50_ doses of imidacloprid and sulfoxaflor against *H. axyridis* adults by simulating a field exposure. Because *H. axyridis* in the field can be simultaneously exposed to insecticide residues through contact and ingestion, respectively, on contaminated-plants and contaminated-prey, we used the methodology proposed by Dai et al. [61]. Briefly, young and unmated *H. axyridis* females (60–72 h old) from the rearing were exposed for three days to insecticide residues of the chosen insecticides at their LC_50_ and LC_20_ on aphid infested cotton plants. Through preliminary observations, twelve pots infested by mixed cotton aphid colonies (more than 2000 aphids per pot) were considered optimal to satisfy for three days the feeding uptake of 20 *H. axyridis* females. Aphid infested cotton pots were sprayed by the insecticidal solutions within a distance of 0.5 m with a 2 L hand sprayer until the solution ran off leaves, and they were left to dry for 1 h in laboratory conditions. Per each replicate, 20 *H. axyridis* young unmated females were exposed to fresh insecticide residues on treated infested cotton plants for 3 days inside a ventilated and screened with fine mesh net. The mortality of *H. axyridis* was recorded after 72 h of exposure. *Harmonia axyridis* individuals were considered dead when they did not react after being touched with a fine brush. Control treatment consisted of untreated infested plants sprayed by distilled water. Five replicates were carried out per each insecticide-concentration and the control.

### 2.5. Sublethal Effect on Longevity and Reproductive Traits

The sublethal impact of the imidacloprid and sulfoxaflor at their LC_20_ and LC_50_ doses, previously calculated for the target pest, was assessed on the longevity, fecundity and fertility of the surviving females from the bioassay “*2.3 Lethal toxicity on Harmonia axyridis*”. Each surviving female was paired with an untreated male of the same age. Each pair of female and male *H. axyridis* was transferred with a fine brush to a ventilated Petri dish (9 cm diameter, 2 cm high) and fed with sterilized *Ephestia kuehniella* Zeller (Lepidoptera: Pyralidae) eggs, since this factitious prey has been recognized as an optimal food source for the laboratory rearing of *H. axyridis* [62]. The *E. kuehniella* eggs were provided daily, the Petri dishes were cleaned daily, and water was offered to the adults for all the treatments. A Z-fold filter paper was also fixed into each Petri dish as an artificial oviposition substrate. Oviposition and egg hatching for each female were recorded for 30 days from the first egg-laying event, while longevity was measured for females until they died. A representative 10% of each fresh egg batch was picked out and isolated in one hole of a 24-hole plate and checked daily to assess the hatch rate until 50 eggs per couple were tested. Between 72 and 139 adult couples were studied per each pesticide-concentration and the control.

### 2.6. Transgenerational Effects on Offspring Survival Rate and Developmental Time

The fresh eggs (≤12 h) of the first batch laid by *H. axyridis* females exposed to insecticide in “*2.3 Lethal Toxicity on H. Axyridis*” were individually transferred with a wet soft fine brush into a ventilated Petri dish (3.5 cm diameter, 1.5 cm high) for hatching (2–3 eggs per couple). New hatchlings were fed with *E. kuehniella* eggs daily, and Petri dishes were cleaned daily. For all treatments, water was supplied through a soaked cotton wad in the Petri dish, and cotton was replaced daily.

The development parameters of larvae were recorded every 24 h. A total of 150 newly hatched larvae were reared per each insecticide-concentration and for the untreated control.

### 2.7. Statistical Analysis

The LC_20_ and LC_50_ values for imidacloprid and sulfoxaflor were estimated by Probit analysis [63] after 48 h exposure to insecticides. The concentration–mortality relationships were considered true when the observed data and the estimated data did not significantly differ (*p* < 0.05).

Levene and Shapiro–Wilk tests were used to check the homogeneity and normality of variance of the dataset that was transformed whenever required. The effects of the two insecticides (factor: insecticide), their LCs (factor: concentration) and their interaction (insecticide x concentration) on the mortality of female adults were analyzed by factorial ANOVA. The longevity of females to different insecticide-concentration exposure was analyzed using the Kaplan–Meier procedure followed by the log rank (Mantel–Cox) test among treatments. The reproductive parameters were analyzed using the ANOVA for the fecundity and fertility data. Differences among the insecticide treatments in the ANOVAs were highlighted by Tukey’s HSD test. Developmental duration of offspring and survival rate of juveniles of *H. axyridis* were analyzed by Kaplan–Meier estimate. Statistical analyses were carried out on SPSS Statistics v. 20.0 (IBM Corp., Armonk, NY, USA).

## 3. Results

### 3.1. Insecticide Baseline Toxicity on Prey

From the log-probit regression analysis, the LC_20_ and LC_50_ values of imidacloprid and sulfoxaflor to *A. gossypii* adults were estimated and listed in Table 2. Observed data fit the log-probit model and no statistically significant deviation from the regression equation was observed. Imidacloprid exhibited the highest toxicity against the cotton aphid laboratory population showing lower LCs values in comparison to sulfoxaflor insecticide (Table 2).

### 3.2. Lethal Toxicity on Harmonia Axyridis

**Figure 1:**
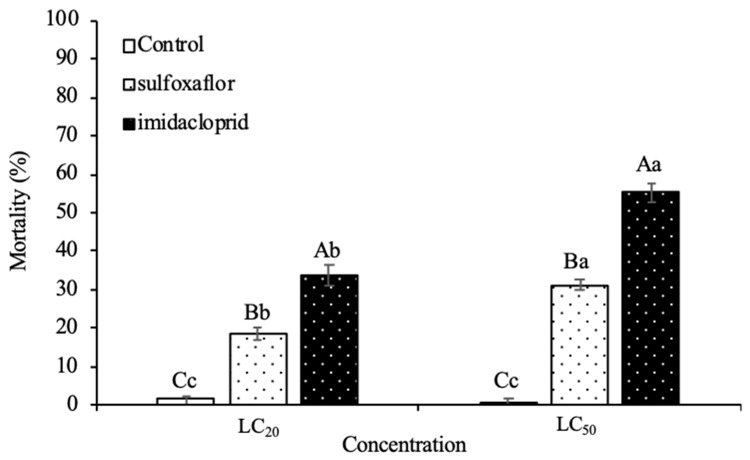
Mean (±SE) of percentage mortality of *Harmonia axyridis* females exposed to imidacloprid and sulfoxaflor and their LC_50_ and LC_20_ estimated for the target pest, *Aphis gossypii*. Columns bearing the different letter (capital letters: within the same concentration regime; lowercase letters: within the same tested insecticide) are significantly different (Tukey’s HSD test for multiple comparisons at *p* < 0.05).

The mortality of the females of *H. axyridis* exposed simultaneously to dry residues by contact on sprayed plants and by ingestion of contaminated prey after 3 days was significantly affected by the insecticides (at the LC_20_ of insecticides, *F*_2,12_ = 60.041, *p* < 0.001; at the LC_50_ of insecticides, *F*_2,12_ = 205.682, *p* < 0.001), the concentration of LC_20_ and LC_50_ (imidacloprid, *F*_2,12_ = 119.465, *p* < 0.001; sulfoxaflor, *F*_2,12_ = 103.258, *p* < 0.001) and their interaction (*F*_5,24_ = 136.693, *p* < 0.001) (Figure 1). Both insecticides and concentrations caused a significantly higher mortality of the exposed predators compared to the control. Namely, LC_50s_ of the two insecticides caused significantly higher mortality than LC_20s_. Compared to mortality caused by sulfoxaflor at LC_50_ (31.20 ± 1.50%), imidacloprid at LC_50_ (55.20 ± 2.33%) was the most harmful insecticide to *H. axyridis* females. In the LC_20_ experiment, the result was similar with the LC_50_ experiment, that is, imidacloprid with a mean mortality of 33.60 ± 2.71% caused a higher lethal effect than sulfoxaflor with an average mortality of 18.40 ± 1.60%.

### 3.3. Influence of Insecticides on Longevity and Fecundity of Females

Statistically significant differences were found in the longevity (*χ*^2^ = 12.139, *df* = 4, *p* = 0.016) of females between the insecticide-concentration combination and control. Moreover, sulfoxaflor LC_20_ significantly increased longevity of female adults. The adult longevity of other treatments showed no significant differences with control treatment; however, imidacloprid at LC_50_ significantly decreased longevity of female adults compare with LC_20_ and LC_50_ doses of sulfoxaflor.

Low lethal concentrations of sulfoxaflor and imidacloprid significantly reduced the percentage of females able to lay eggs. The influence of sulfoxaflor on female fertility rate (*χ*^2^ = 52.217, *df* = 4, *p* < 0.001) at the same sublethal concentration was less than that of imidacloprid, but there were no statistically significant differences between them. Meanwhile, the fecundity per female was significantly reduced to all sublethal concentrations (*F*_4,342_ = 5.670, *p* < 0.001) except for sulfoxaflor LC_20_ in comparison to the control (Table 3).

### 3.4. Effect of Insecticides on Survival Rate and Developmental Time of Offspring

The effect of the LC_20_ and LC_50_ concentrations of imidacloprid and sulfoxaflor on egg hatchability, larval survival and adult emergence of F_1_ generation are presented in Table 4. The egg hatchability (*F*_4,342_ = 8.254, *p* < 0.001) of *H. axyridis* significantly decreased at LC_50s_ of imidacloprid and sulfoxaflor compared to the control; however, LC_20_ of imidacloprid and sulfoxaflor showed no significant differences with control. No significant differences were found between imidacloprid and sulfoxaflor at the same sublethal concentration. The sublethal concentration of imidacloprid and sulfoxaflor significantly affected the survival of *H. axyridis* larvae (overall comparisons, *χ*^2^ = 19.104, *df* = 4, *p* = 0.001). In *Harmonia axyridis* larvae, F_1_ generation of female parents exposed to imidacloprid LC_50_ led to the lowest survivorship, while sulfoxaflor LC_20_ induced the highest survivorship. According to analysis of pupal survival (*χ*^2^ = 8.248, *df* = 4, *p* = 0.083), the sublethal concentration of two insecticides led to a decrease in pupal survival in comparison to control, but no significant differences were found between the two insecticides and the two concentrations.

The influence of imidacloprid and sulfoxaflor at the two low lethal concentrations on developmental time of the F1 generation (egg, larva and pupa) of *H. axyridis* is listed in Table 5. LC_20_ of sulfoxaflor caused no significant impact on the developmental time of egg (*χ*^2^ = 75.812, *df* = 4, *p* < 0.001) and larva (*χ*^2^ = 102.805, *df* = 4, *p* < 0.001), and other insecticide treatments significantly lengthened the developmental time of egg and larva in comparison to control treatment. No significant differences were found in the pupal developmental time (*χ*^2^ = 16.703, *df* = 4, *p* = 0.002) between the treatments and control, but sulfoxaflor at LC_50_ significantly prolonged the developmental time of pupa compared to LC_20_.

Imidacloprid and sulfoxaflor at the LC_20_ and LC_50_ doses significantly lengthened developmental time (1st instar, *χ*^2^ = 38.055, *df* = 4, *p* < 0.001; 2nd instar, *χ*^2^ = 24.727, *df* = 4, *p* < 0.001; 3rd instar, *χ*^2^ = 64.632, *df* = 4, *p* < 0.001; 4th instar, *χ*^2^ = 76.762, *df* = 4, *p* < 0.001) of instars, especially for the 1st, 3rd and 4th instars (Table 6).

## 4. Discussion

The present study provides evidence of acute and sub-lethal effects of imidacloprid and sulfoxaflor against adults female *H. axyridis*. Transgenerational sub-lethal effects of these insecticides were also found in the progeny of surviving females mated with untreated males.

Dai et al. reported that imidacloprid (0.503 ppm, 3.186 ppm) and sulfoxaflor (0.397 ppm, 2.000 ppm) have no significant lethal effect on female *H. axyridis* [61]. Here the results on acute toxicity are not in accordance with Dai et al. [61], because LC_20_ and LC_50_ values are higher in the present study. Such differences in toxicity toward the target pest may be due to the different susceptibility of the *A. gossypii* strains used in the various experiments, with the population of the present study being less susceptible to the two chemicals compared to the other study.

In our study, the results showed no significant effect of imidacloprid with LC_20_ and LC_50_ concentrations and sulfoxaflor with LC_50_ concentrations on the longevity of *H. axyridis* females. However, sublethal concentration (LC_20_) of sulfoxaflor significantly increased longevity of *H. axyridis*. This was probably because the sulfoxaflor was very safe at LC_20_ concentration, and the number of spawned eggs of sulfoxaflor (LC_20_) treatment were less than the control. *H. axyridis*, treated by sulfoxaflor (LC_20_), can spend more energy to maintain non-reproductive life activities. Skouras et al. reported that imidacloprid at sublethal concentrations reduced longevity of *Hippodamia variegata* (Coleoptera: Coccinellidae) [64]. Papachristos et al. reported a similar result, which demonstrated that low concentration of imidacloprid can reduce longevity of *Hippodamia undecimnotata* (Coleoptera: Coccinellidae) [65]. However, in agreement with our findings, Rahmani et al. reported that sublethal concentrations of thiamethoxam had no significant negative effect on longevity of *H. variegate* when the 3rd instar larvae were exposed [66]. The results of insecticide effects on longevity of ladybird beetles were variable, which may be influenced by the molecular structure of the insecticide and the treatment method.

Sublethal concentrations (LC_20_ and LC_50_) of imidacloprid and sulfoxaflor had significant negative influences on adult fecundity and egg hatchability of *H. axyridis* when the 3-day-old females were exposed. These results are in accordance with Jiang et al., Yu et al. and Xiao et al. [67,68,69]. Jiang et al. reported that thiamethoxam at LC_10_ and 0.1 × LC_10_ had significant negative effects on survival rate, adult emergence rate, fecundity and egg hatchability of *Coccinella septempunctata* L. (Coleoptera: Coccinellidae). Yu et al. reported that imidacloprid, at sublethal concentrations, might impair adult emergence and reproduction of *C. septempunctata*. Xiao et al. found sublethal effects on the reproduction of *C. septempunctata* residually exposed to 10% of LC_5_ and LC_5_ of imidacloprid.

The sublethal and transgenerational effects of insecticides have been overlooked in many cases, even though they may negatively affect beneficial arthropod communities and have a significant impact on ecological services [39,48,70,71]. Moreover, we evaluated the transgenerational effects of imidacloprid and sulfoxaflor on survival rate and developmental time of *H. axyridis*. Our results demonstrated that the rates of hatching, larvae survival and emergence significantly decreased, and the egg and instar stages of the F1 generation could apparently be prolonged, when its parental generation F0 were exposed to imidacloprid and sulfoxaflor at the LC_20_ and LC_50_ concentrations. Xiao et al. found that the progeny of these individuals of *C. septempunctata* had a lower demographical growth compared to the untreated control (transgenerational effects) [69]. Similarly, thiamethoxam applied to cotton seed influenced larvae or adults of *Chrysoperla externa* and *H. axyridis*, which also reduced juvenile survival of individuals in the following generation [72].

Overall, the present study disclosed that low concentrations of sulfoxaflor and imidacloprid can cause acute toxicity and impair the biological parameters of a parental generation of *H. axyridis* and its offspring by contact on plant and ingestion of contaminated prey. However, sulfoxaflor caused lower mortality and affected less *H. axyridis* biological traits at intra- and transgenerational levels than imidacloprid, thus proving to be a safer compound in the laboratory. Nonetheless, further specific long-term studies are necessary to reveal the mechanisms behind the effect of low insecticide concentrations on *H. axyridis*.

Our outcomes stress also the need to include in-depth biological evaluations into pesticide risk-assessment schemes towards beneficial arthropods. Numerous studies recognized the importance of addressing studies on pesticide sublethal effect [73,74]. Similarly, the combination of chemical stressors with different thermal regimes can be assessed towards beneficial predators considering the ever-changing environment as projected by the current global warming scenario [75].

## 5. Conclusions

Experimental results indicated that low concentrations of sulfoxaflor and imidacloprid both had acute toxicity against *H. axyridis* and negative sublethal effects on its population development. Therefore, sulfoxaflor and imidacloprid should be cautiously used in IPM programs against *A. gossypii*. Otherwise, the ecological service and effectiveness of harlequin ladybirds will be reduced. Compared with the traditional nicotinic insecticide imidacloprid, sulfoxaflor appears safer for the harlequin ladybird, which has less influence on the survival rate of ladybird adults and progeny and their fecundity. The results of this study will further improve our understanding of the effects of insecticide residues on ladybird population development and provide a basis for more scientific and efficient measures to manage *A. gossypii* and protect coccinellid beetles.

## Figures and Tables

**Table 1:** Formulation, field-recommended concentration and manufacturer of two insecticides tested for their side effects on *Harmonia axyridis*.

Chemical Classes	Active Ingredient	IRAC Group	Formulation		Field-Recommended Dose (g a.i. ha^−1^/ppm)	Manufacturer
			% of a.i.	Type		
Neonicotinoids	imidacloprid	4A	70	WG	31.5/70	Bayer Crop Science (China) Company Limited
Sulfoximines	sulfoxaflor	4C	22	SC	49.5/110	Dow AgroSciences (Repacking units: Jiangsu Suzhou Jiahui Chemical Company Limited)

Note: a.i. = active ingredient. WG = wettable granules. SC = suspension concentrate. ppm = mg a.i. L^−1^.

**Table 2:** Baseline toxicity results following contact exposure of two insecticides against *Aphis gossypii* adults.

Insecticide	Regression Equation of Toxicity	*χ* ^2^	n	*df*	*p*	LC_20_ (ppm) (95% Fiducial Limits)	LC_50_ (ppm) (95% Fiducial Limits)
Imidacloprid	Y = 2.670 ± 1.561X	14.39	30	28	0.984	3.93 (2.85–5.07)	13.62 (11.34–16.15)
Sulfoxaflor	Y = 1.941 ± 1.742X	10.53	30	28	0.999	5.56 (4.37–6.77)	16.90 (14.44–19.80)

**Table 3:** Mean (±SE) values of longevity and fecundity of *Harmonia axyridis* females exposed to imidacloprid and sulfoxaflor and their LC_50_ and LC_20_ estimated for the target pest, *Aphis gossypii*. Values followed by different letters, within the same column, indicate significant differences at *p* < 0.05 level.

Treatment	Longevity (d)	Fecundity in 30 Days (Eggs Per Female)	Egg-Laying Females (%)
Control	102.01 ± 5.81 bc	694.77 ± 37.96 a	97.22 ± 1.94 a
imidacloprid LC_20_	111.10 ± 8.15 abc	531.81 ± 44.80 bc	72.55 ± 4.42 bc
imidacloprid LC_50_	92.91 ± 7.21 c	432.75 ± 41.18 c	52.46 ± 4.52 d
sulfoxaflor LC_20_	117.94 ± 8.83 a	595.55 ± 37.42 ab	79.38 ± 4.22 b
sulfoxaflor LC_50_	115.66 ± 8.97 ab	507.71 ± 42.32 bc	60.00 ± 4.67 cd

**Table 4:** Mean (±SE) values of egg-hatchability, larval and pupal survival of the offspring of *Harmonia axyridis* females exposed to imidacloprid and sulfoxaflor and their LC_50_ and LC_20_ estimated for the target pest, *Aphis gossypii*. Values followed by different letters, within the same column, indicate significant differences at *p* < 0.05 level.

Treatment	Egg Hatchability (%)	Larval Survival (%)	Pupal Survival (%)
Control	81.81 ± 2.92 a	82.67 ± 3.09 ab	99.19 ± 0.80 a
imidacloprid LC_20_	71.75 ± 2.93 abc	76.67 ± 3.45 bc	94.78 ± 2.07 b
imidacloprid LC_50_	61.26 ± 3.39 c	68.00 ± 3.81 c	99.02 ± 0.98 ab
sulfoxaflor LC_20_	76.86 ± 2.23 ab	86.00 ± 2.83 a	97.67 ± 1.33 ab
sulfoxaflor LC_50_	62.33 ± 3.91 c	71.33 ± 3.69 c	94.39 ± 2.22 b

**Table 5:** Mean (±SE) values of stage-specific developmental duration of the progeny of *Harmonia axyridis* females exposed to imidacloprid and sulfoxaflor and their LC_50_ and LC_20_ estimated for the target pest, *Aphis gossypii*. Values followed by different letters, within the same column, indicate significant differences at *p* < 0.05 level.

Treatment	Egg (d)	Larva (d)	Pupa (d)
Control	2.90 ± 0.03 c	12.40 ± 0.12 c	4.29 ± 0.04 ab
imidacloprid LC_20_	3.00 ± 0.01 b	13.69 ± 0.22 b	4.18 ± 0.04 b
imidacloprid LC_50_	2.99 ± 0.02 bc	14.77 ± 0.33 a	4.35 ± 0.04 ab
sulfoxaflor LC_20_	2.91 ± 0.03 bc	12.76 ± 0.15 c	4.25 ± 0.05 b
sulfoxaflor LC_50_	3.19 ± 0.03 a	14.50 ± 0.24 ab	4.44 ± 0.05 a

**Table 6:** Mean (±SE) values of stage-specific larval developmental duration of the progeny of *Harmonia axyridis* females exposed to imidacloprid and sulfoxaflor and their LC_50_ and LC_20_ estimated for the target pest, *Aphis gossypii*. Values followed by different letters, within the same column, indicate significant differences at *p* < 0.05 level.

Treatment	1st Instar (d)	2nd Instar (d)	3rd Instar (d)	4th Instar (d)
Control	2.16 ± 0.04 c	2.11 ± 0.06 ab	2.68 ± 0.06 b	5.49 ± 0.07 c
imidacloprid LC_20_	2.65 ± 0.10 a	2.13 ± 0.07 ab	2.69 ± 0.07 b	6.23 ± 0.14 b
imidacloprid LC_50_	2.61 ± 0.12 ab	2.30 ± 0.09 a	3.02 ± 0.09 a	6.76 ± 0.15 a
sulfoxaflor LC_20_	2.33 ± 0.05 bc	1.88 ± 0.05 b	2.47 ± 0.06 b	6.09 ± 0.14 b
sulfoxaflor LC_50_	2.17 ± 0.04 c	2.25 ± 0.07 a	3.24 ± 0.10 a	6.95 ± 0.16 a

## Data Availability

The data presented in this study are available on request from the corresponding authors.
